# The Capacity of Cognitive Control Estimated from a Perceptual Decision Making Task

**DOI:** 10.1038/srep34025

**Published:** 2016-09-23

**Authors:** Tingting Wu, Alexander J. Dufford, Melissa-Ann Mackie, Laura J. Egan, Jin Fan

**Affiliations:** 1 Department of Psychology, Queens College, The City University of New York, Queens, NY 11367, USA; 2 Departments of Psychiatry, Icahn School of Medicine at Mount Sinai, New York, NY 10029, USA; 3 Department of Psychology, The Graduate Center, The City University of New York, New York, NY 10016, USA; 4 Neuroscience, Icahn School of Medicine at Mount Sinai, New York, NY 10029, USA

## Abstract

Cognitive control refers to the processes that permit selection and prioritization of information processing in different cognitive domains to reach the capacity-limited conscious mind. Although previous studies have suggested that the capacity of cognitive control itself is limited, a direct quantification of this capacity has not been attempted. In this behavioral study, we manipulated the information rate of cognitive control by parametrically varying both the uncertainty of stimul measured as information entropy and the exposure time of the stimuli. We used the relationship between the participants’ response accuracy and the information rate of cognitive control (in bits per second, bps) in the model fitting to estimate the capacity of cognitive control. We found that the capacity of cognitive control was approximately 3 to 4 bps, demonstrating that cognitive control as a higher-level function has a remarkably low capacity. This quantification of the capacity of cognitive control may have significant theoretical and clinical implications.

The human brain processes a multitude of information in multiple cognitive domains under time constraints. The amount of domain-specific information that can be processed during a certain period of time has an estimated capacity ranging from 2 to 60 bits per second (bps) for attention, decision-making, perception, motion, and language, and up to 10^6 ^bps for sensory processing^1^,^2^,^3^,^4^,^5^,^6^,^7^. However, the conscious mind can only handle a portion of higher-order information at a time. Therefore, cognitive control is essential to efficiently select and prioritize important information to reach the conscious mind and to act on that information^8^,^9^. Although it has been proposed that the capacity of cognitive control (CCC) is limited^8^,^10^,^11^, a quantification of CCC is still lacking. In the past, the quantification of limited working memory capacity, as the “magic” 7 ± 2^1^, has fundamentally influenced the fields of psychology and cognitive science. Quantification of the CCC, even in a domain such as perceptual decision-making as a specific case, may advance our understanding of the mechanisms and upper-bound limit of cognitive control.

Information theory^12^ provides a theoretical and mathematical framework for the quantification of cognitive processes^1^,^13^,^14^,^15^,^16^. Under this framework, the uncertainty of information is quantified as the information entropy in unit of bits for a given channel, and the information rate is the average amount of information transmitted per unit of time. According to the noisy-channel coding theorem, channel capacity is defined by the upper bound of the information rate at which information can be transmitted with an arbitrarily small error probability. The accuracy of information transmission decreases when the required transmission rate exceeds the capacity. Therefore, the capacity of a “channel” for cognitive processing can be estimated if there is a manipulation of information rate to challenge the cognitive processing capacity and the corresponding performance is measured.

In this study, we parametrically manipulated the rate of information processing that requires cognitive control within a wide range to assess participants’ performance when the CCC is challenged to varying degrees. The entropy of the to-be-controlled information was manipulated using the majority function task (MFT)^17^, which has been demonstrated as a valid paradigm to study cognitive control with converging evidence of the correlation between MFT performance and flanker conflict effect^18^. The MFT requires the process of reconciling conflicting information, which is also a psychological property of the flanker task, but with greater engagement of cognitive control to coordinate mental operations for perceptual information processing and decision-making. Additionally, we also varied the exposure time (ET) of the stimuli using a backward masking approach as a manipulation to engage variable information processing rate. As the required information rate begins to exceed participants’ CCC, the response accuracy will begin to decrease. The relationship between response accuracy and required information rate depends on the CCC. By fitting a model with predicted accuracy as a function of CCC to empirical behavioral performance in terms of accuracy, we could obtain an optimized CCC value as the estimate of the CCC.

## Material and Methods

### Participants

Thirty-two adult volunteers participated in the study. Two participants were excluded from further analysis due to low task accuracy (3 standard deviations (SD) lower than the group mean in some conditions). The final sample size was 30 (19 females and 11 males; mean age: 24.8 years, range: 18–38 years). This study was approved by the Institutional Review Board (IRB) of Queens College of The City University of New York and conducted in accordance with the guidelines of the IRB. Written informed consent was obtained from each participant.

### The backward masking majority function task

**Figure 1:**
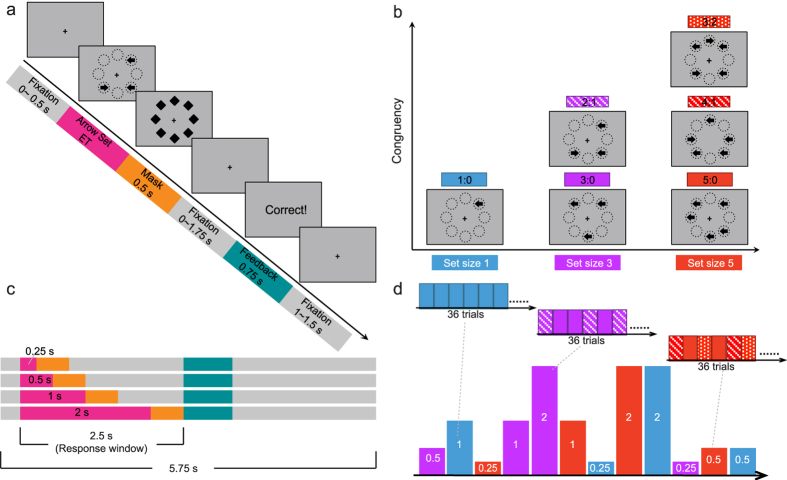
Schematic of the backward masking majority function task (MFT-M). (**a**) The sequence of a trial in the MFT-M (2:1 condition). Stimuli events are indicated by color-coding. Participants were required to report the majority of arrow directions (left or right). (**b**) Possible congruency ratios (majority : minority) of arrow sets. Set size represents the total number of arrows. (**c)** Timeline of the stimuli under different stimulus exposure time (ET, in seconds). Duration of each event is illustrated by the length of each color bar. A response was required within a 2.5 s response window and the total length of each trial was 5.75 s. (**d**) The block structure and order. There were 12 task blocks in this task (lower panel), corresponding to 12 possible combinations of set size (represented by the color of a block) and ET (represented by the height of a block). Each task block consisted of 36 trials, with equal proportion of all possible congruencies under the corresponding set size (represented by color patterns in upper panel). The order of blocks was randomized between participants.

The backward masking majority function task (MFT-M) was based on the MFT^17^. All parameters in the MFT-M were identical to our previous studies using the MFT^17^ except that the ET of the arrow set was manipulated by varying stimulus duration and applying backward masking. The mask consisted of eight solid diamond shapes presented at the same eight positions at which the arrows could appear. In the MFT-M, groups of arrows with set sizes of 1, 3, and 5 were randomly presented at eight possible locations arranged as an octagon centered on a fixation cross (Fig. 1a,b). Each arrow pointed either left or right and all arrows were presented simultaneously. The congruency of the arrow set refers to the ratio between the majority and minority direction of arrows, which could be 1:0, 3:0, 2:1, 5:0, 4:1, or 3:2. The length of the arrow and the diameter of each diamond shape was 0.37° of visual angle. The radius from the fixation cross to the center of an arrow subtended approximately 1.5° of visual angle. This small visual angle was used to avoid excessive eye movement.

At the beginning of each trial, there was a variable fixation period of 0 to 0.5 s. Following this fixation period, an arrow set was presented with a variable ET followed by a mask for 0.5 s. The ET was 0.25, 0.5, 1, or 2 s (Fig. 1c). The arrow set remained on the screen for the duration of the ET period. After the offset of the mask, there was a variable post-stimulus fixation period for 0 to 1.75 s to make the total duration of the arrow set, mask, and post-stimulus fixation together 2.5 s. Participants were instructed to make a response as accurately and quickly as possible to indicate the direction in which the majority of the arrows pointed by pressing one of two buttons with the index or middle finger of their right hand. The response accuracy was emphasized above the reaction time (RT). Responses had to be made within a 2.5 s period from the onset of the target. Participants were instructed to guess when they failed to find the majority direction and to respond in all trials. Following this 2.5 s period, feedback was presented for 0.75 s to inform participants whether their response in the current trial was correct. At the end of each trial, there was a variable post-feedback fixation period for 1 to 1.5 s to make the total duration of each trial as 5.75 s.

The task consisted of 12 blocks with each block comprised of one of the combinations of the set sizes and ETs (Fig. 1d). The set sizes and ETs were varied between blocks but not trial-by-trial to reduce baseline uncertainty. The presentation of the blocks was in a random order and each block consisted of 36 trials. The presentation of the trials within each block was also in a random order. Within each block, the number of trials in each congruency condition was identical (with equal number of correct responses for left and right): 36 trials under 1:0 condition in a one-arrow block, 18 trials under 3:0 and 18 trials under 2:1 conditions in a three-arrow block, and 12 trials under 3:2, 12 trials under 4:1, and 12 trials under 5:0 conditions in a five-arrow block. At the beginning and end of each block there was a 3 s fixation period. Each block lasted 213 s. Participants completed two sessions of this task separated by a range of 1 to 14 days. The two sessions were identical except for block order. During each session, 432 trials in total were presented and the task duration was approximately 43 minutes. The data from the two sessions were combined for a more reliable estimation of the CCC.

The task was run on a PC using E-Prime software (Psychology Software Tools, Pittsburgh, PA). It was first explained to the participants verbally. Once an understanding of the task was demonstrated, participants completed a five-minute practice session before performing the first experimental session.

### Analyses of the task performance

Trials with no response within the response window (2.5 s from the target onset) were treated as error trials and were excluded from the RT analysis. Trials with RT exceeding ± 3 SD of the mean RT in each condition were considered as outliers and were removed from further analysis of RT. Mean and SD of RT under each condition were calculated based on the remaining trials for each participant. Accuracy for each condition was computed as the percentage of trials with correct responses.

The group mean and SD of the accuracy and RT for each condition of the final sample were calculated. Two 2 (Session: 1, 2) × 4 (ET: 0.25, 0.5, 1, 2 s) × 6 (Ratio, and later on also referred as Entropy based on the associated entropy values: 1:0, 3:0, 5:0, 2:1, 4:1, 3:2) repeated measured ANOVAs were conducted on the accuracy and RT separately (see below for the definition of the Entropy factor). Bonferroni correction was used to correct for multiple comparisons in the post-hoc comparisons.

### Grouping search algorithms adopted during the performance of the task

**Figure 2:**
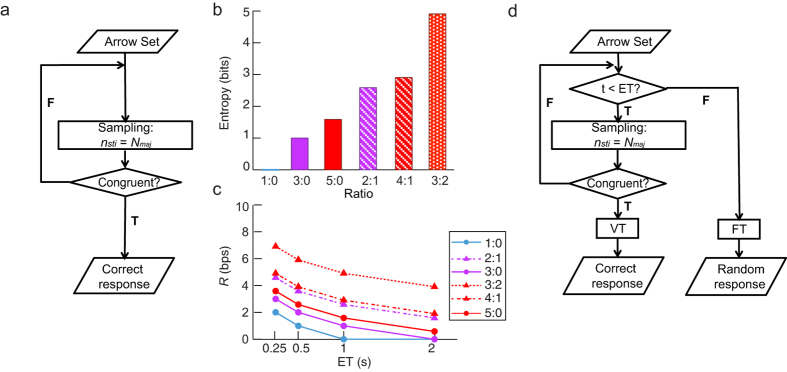
Mental algorithm underlying the MFT-M. (**a**) An illustration of the grouping search strategy. Participants repeatedly sample stimuli with a majority size until a congruent sample is obtained. *n*_*sti*_: number of stimuli in one sample. *N*_*maj*_: majority size. T: true. F: false. The average entropy (**b**) and average information rate (*R*) (**c**) in different conditions of the MFT-M, estimated according to the grouping search algorithm. (**d**) An illustration of the time-constrained grouping search algorithm. The sampling process is voluntarily terminated (VT) when a congruent sample is obtained, or forcedly terminated (FT) when stimuli have disappeared. A VT will lead to a correct response, while a FT will lead to a random response.

The grouping search, which has been indicated as the most plausible algorithm to explain participants’ strategy in this task^17^,^19^,^20^, refers to a strategy in which participants repeatedly sample stimuli as a group with a majority size (over half of the total set size: 1, 2, and 3 for set sizes of 1, 3, and 5, respectively). Each sampling is made randomly and independent of the others. The sampling process is terminated only after a congruent sample is obtained, i.e., all arrows in a sampled group are pointing to the same direction. A response is subsequently made based on this congruent sample. See Fig. 2a for an illustration of the grouping search strategy.

The average information entropy (in unit of bit) of each ratio condition can be calculated as the *log*_*2*_ transform of the average number of arrows that need to be scanned to obtain a congruent sample, which can be computed as the majority size (*N*_*maj*_) divided by the probability of obtaining a congruent sample from one sampling attempt (*P*_*group*_): entropy = *log*_*2*_(*N*_*maj*_/*P*_*group*_). For a given condition, the *P*_*group*_ can be calculated as:


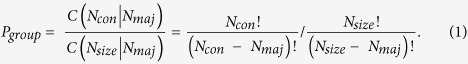


Here *N*_*size*_ is the set size and *N*_*con*_ is the number of arrows pointing to the majority direction. According to Equation 1, the *P*_*group*_ is 1.00, 1.00, 0.33, 1.00, 0.40, and 0.10 for 1:0, 3:0, 2:1, 5:0, 4:1, and 3:2 ratio conditions respectively, corresponding to entropy of 0, 1.00, 2.58, 1.58, 2.91 and 4.91 bit(s) (Fig. 2b). The average information rate (R) of each condition was calculated as the *log*_*2*_ transformation of the average number of to-be-scanned arrows in each second: 
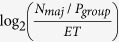
, which represents the bits to-be-processed in a second, varied from 0 to 6.91 bps (Fig. 2c). Because the number of to-be-scanned arrows in one second 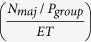 was less than one in the ratio = 1:0 and ET = 2 s condition, which corresponds to a negative information rate, we set the R as 0 bps for this condition assuming that scanning less than one arrow is meaningless for detecting arrow direction.

### MFT-M response accuracy as a function of the capacity of cognitive control

Because the ET of stimuli in the MFT-M is restricted with the backward masking, we assume that the sampling process can only be implemented while the stimuli are still displayed on the screen. The sampling process can be either voluntarily terminated (VT) when a congruent sample is obtained or forcefully terminated (FT) when the stimuli disappear before a congruent sample is obtained. The responses of VT trials should be relatively accurate, while responses of FT trials would be made randomly because we instructed participants to guess if they failed to find the majority direction. See Fig. 2d for the illustration of this time-restricted grouping search algorithm.

For a given condition, the probability of VT (*P*_*VT*_) can be calculated as


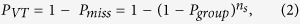


where *P*_*miss*_ is the probability that no congruent sample is detected from a certain number of scanned samples (*n*_*s*_). The *n*_*s*_ is determined by CCC and ET, which can be calculated as


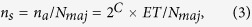


where *n*_*a*_ is the number of scanned arrows, and *n*_*a*_ and *n*_*s*_ are not necessarily integers. *C* is a free parameter to denote the CCC. Here *C* = *log*_*2*_ (*n*_*a*_/ET), as *log*_*2*_ transformation of the number of to-be-scanned arrows in a unit of time (i.e., per second). Therefore, it is the bits to-be-processed per second (bps). For the explanation of the *log*_*2*_ (*n*_*a*_) to information entropy in bit transformation, please refer to our previous study using the MFT^17^. For a given condition, a higher *C* and a longer ET would lead to a higher probability of VT.

Both VT and FT may occur in the MFT-M, and the expected response accuracy (*E*[*accuracy*]) can be calculated as


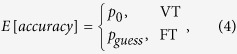


where *p*_*0*_ is the baseline response accuracy basing on a congruent sample, which can be computed as the average accuracy across all of the congruent conditions (1:0, 3:0, and 5:0, averaged across ETs), and *p*_*guess*_ is the chance level for guessing, which is 50% because there are two choice alternatives with equivalent probability (left and right). The *E*[*accuracy*] varies as a function of *P*_*VT*_^3^,^21^:


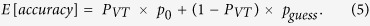


Therefore, the response accuracy varies as a function of Entropy, *C*, and ET:


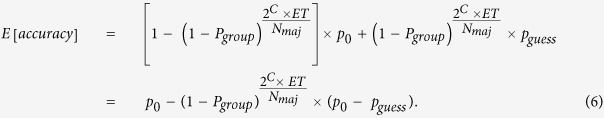


As the amount of information increasingly exceeds CCC, FT will occur more often and response accuracy will reduce towards the chance level.

### Simulation analysis

**Figure 3:**
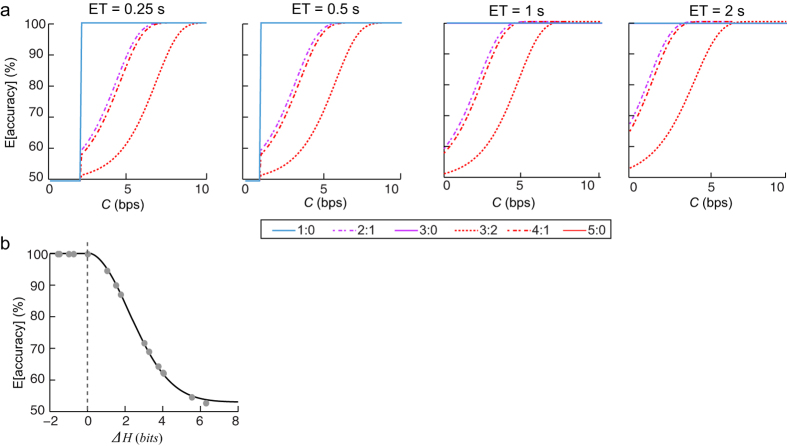
Results of the simulation analysis. (**a**) The predicted accuracy (*E*[*accuracy*]) as a function of the capacity parameter (labeled as *C*). Note: the lines that represent the predicted accuracy in three congruent conditions (1:0, 3:0, and 5:0) overlapped across all ET because their *E*[*accuracy*] values were equivalent. (**b**) An illustration of the *E*[*accuracy*] as a function of *ΔH*, under a given *C* set as 3 bps. Here *ΔH* is the difference between can-be-transmitted and to-be transmitted information within each ET. The *E*[accuracy] was perfect when the to-be-processed information was lower than the can-be-transmitted information, and then deceased when the to-be-processed information exceeded the can-be-transmitted information until it reached chance level.

To visualize the influence of *C*, ratio, and ET on response accuracy, we conducted a simulation analysis based on Equation 6, with *P*_*group*_, *N*_*maj*_, *ET*, and *C* as the parameters. We varied *C* values from 0 to 10 bps, corresponding to the range of information rate of the task, with a step length of 0.1 bps. The baseline accuracy *p*_*0*_ was 100%. Other parameters were determined by the arrow ratio and ET in each condition. We plotted the relationship of predicted response accuracy on *C* under each condition. See Fig. 3a for the *E*[*accuracy*] corresponding to a set of *C* values ranging from 0 to 10 bps. The *E*[*accuracy*] increased as the *C* values increased, until reaching 100% at a certain *C* value. The inflection points of the predicted accuracy as a function of the *C* curve were associated with larger *C* values in the conditions when the entropy was higher and ET was shorter, reflecting the difficulty of conditions.

In addition, as mentioned above, the more information that exceeds the capacity the lower the accuracy. To visualize this effect, we plotted the relationship of predicted accuracy on *ΔH*, which was defined as the difference between can-be-transmitted (log_2_(*na*)) and to-be transmitted (*H*_*max*_) information within *ET*, under a given *C* as 3 bps. Here we defined the *H*_*max*_ as the information that needs to be processed to reach relatively high response accuracy (e.g., 99%). According to Equations 2 and 5 this maximum attempt of scanning to achieve this accuracy (*n*_*s*_’) can be calculated for each condition as


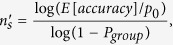


in which the *E*[*accuracy*] was set as 0.99 and *p*_*0*_ was set as 1. The *H*_*max*_ can be computed as *log*_*2*_ (*n*_*s*_’ × *N*_*maj*_). The predicted accuracy was 100% when *H*_*max*_ was lower than information that can be transmitted, and declined when *H*_*max*_ exceeded the information that can be transmitted and moves toward chance level as *H*_*max*_ increases (Fig. 3b).

### Alternative models of mental algorithms

#### Varied chance level in the grouping search model

We also tested whether a varied chance level (*P*_*guess*_) under different Entropy conditions would improve the fitting of the time-restricted grouping search model. In the null model, the chance level (*P*_*guess*_) was a constant of 50%. In the alternative model with varied chance level, *P*_*guess*_ was the ratio between *N*_*maj*_ and *N*_*size*_, which means that participants made responses based on one of the scanned arrows.

#### Varied processing time in the grouping search model

According to the empirical results (see Results section), the averaged RTs in the 2 s ET conditions were shorter than the ET without perfect response accuracy, indicating that participants might not sufficiently use the whole ET to process the information to attain maximum response accuracy. Therefore, in this alternative model, we used the lower value compared RT and ET in each condition to replace the ET terms in Equation 6. Here RTs in trials with incorrect responses were also included, because we assumed that in these trials participants also followed the grouping search strategy but did not obtain any congruent sample before making the responses. In addition, for the other ET conditions (0.25, 0.5, and 1 s), the averaged RT was longer than ET when the accuracy was below 90%. We conducted an additional analysis by using these conditions to re-estimate the CCC and compared the results with the results from the null model.

#### Exhaustive search and self-terminating search

We assumed that the mental algorithm adopted by participants was the grouping search, as confirmed in our previous study^17^. However, it could be argued that under time constraint a different mental algorithm might be employed. For example, the exhaustive search (i.e., scanning all the arrows individually and then returning the majority direction) or self-terminating search (i.e., continually scanning arrows sequentially until the majority can be determined) may be used. Therefore, we compared alternative models for these mental algorithms to the grouping search model.

For the exhaustive search model, the *P*_*VT*_ is calculated as


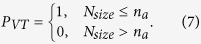


For the self-terminating search model, the *P*_*VT*_ is calculated as


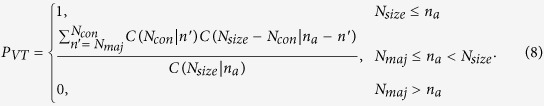


Note, each arrow was considered as a binary number in these two models, which is equivalent to 1 bit. Therefore, the *n*_*a*_ here equals to *C × *ET. The *E*[*accuracy*] in each condition was calculated using Equation 5 based on the *P*_*VT*_ for the exhaustive search and self-terminating search model, respectively. Here the *P*_*guess*_ was 50% for these two models.

### Estimation of the capacity of cognitive control and model comparison

According to Equation 6, for a given *C* value, we can predict the response accuracy in each condition. The performance of prediction can be evaluated using the likelihood (*L*) between predicted and empirical accuracy across all conditions, using the binomial likelihood function:


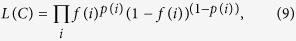


where *f(i)* and *p(i)* are the empirical and the predicted accuracy, respectively, in condition *i*. Higher likelihood indicates a better fit to empirical data by the model. The estimated capacity of cognitive control (*E*[*CCC*]) is the optimal *C* value, which makes best prediction for the empirical response accuracy:


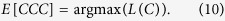


The maximum value of *L* was searched using a range of *C* from 0 to 9.03 bps corresponding to the range of maximum to-be-processed information rate in the MFT-M, calculated as *log*_*2*_(*n*_*s*_’/ET).

The *E*[*CCC*] was first estimated for each participant based on response accuracy in each condition and then its group-mean was calculated. The 95% confidence intervals (CI) of this group-mean *E*[*CCC*] were estimated by the bootstrapping approach because of the relatively small sample size in the current study. The bootstrap sample was created based on sampling with replacement of *E*[*CCC*] values from 30 participants for 10,000 iterations. The 95% CI was determined from the bootstrap sample using the bias corrected and accelerated percentile method.

For model comparison between group search (null model) and alternative models motioned above, the *E*[*CCC*] was estimated for each model. The Bayesian information criterion (*BIC*) was calculated for the predicted and empirical accuracy for each model. A lower *BIC* value indicates better model fitting. The difference of *BIC* values (*ΔBIC*) between this alternative model and the null model was also calculated. Model comparison was conducted for each participant and also for group-average response accuracy.

### Reliability of the estimation of capacity of cognitive control

Reliability of the estimation was only tested for the optimal mental algorithm (grouping search with 50% chance level, see Results). Because we used the average performance across two test sessions, Session 1 and Session 2 were treated as two half sessions and the split-half reliability was calculated as 2*r*/(1 + *r*), where *r* was the correlation coefficient between the two sessions.

## Results

### Response accuracy and RT as a function of entropy and exposure time

**Figure 4:**
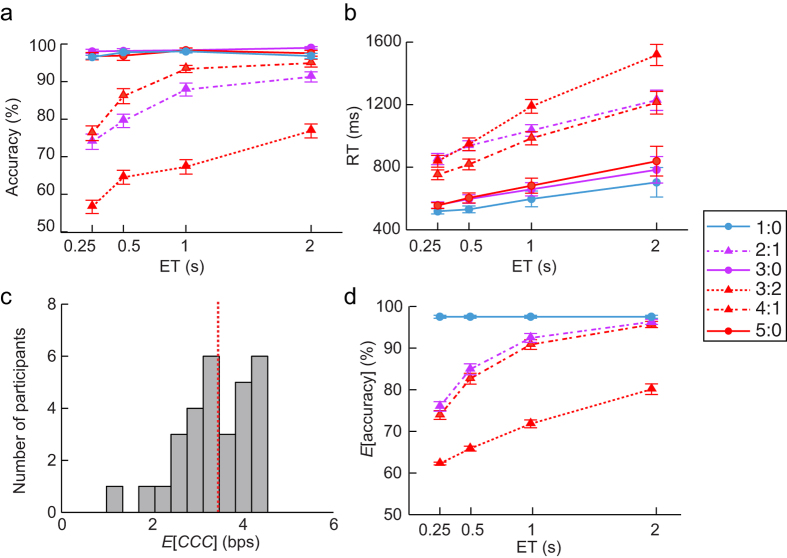
Empirical and predicted behavioral performance, and estimated capacity of cognitive control (*E*[*CCC*]). Group-averaged accuracy (**a**) and reaction time (RT) (**b**) varied as a function of the ET and the entropy. (**c**) Histogram of the distribution of *E*[*CCC*] of the participants. The mean of the distribution is indicated by the intersection of the red dashed line with the x-axis. (**d**) Predicted accuracy (*E*[accuracy]) using the *E*[*CCC*], Entropy, and ET. The lines that represent the predicted accuracy in three congruent conditions (1:0, 3:0, and 5:0) overlapped because of the ceiling effects in these conditions. Error bars represent standard error.

Table 1 shows the group mean and standard error (SE) of response accuracy and RT under each condition. We found that the response accuracy was approximately perfect in the three congruent conditions (1:0, 3:0, and 5:0) across all ETs, but declined as a function of entropy and the reciprocal of ET, and dropped towards chance level as the information rate increased in incongruent conditions (2:1, 4:1, and 3:2) (Fig. 4a). The ANOVA revealed that the main effect of Session was significant, *F*(1, 29) = 26.33, *p* < 0.001, with more accurate performance in Session 2 (M = 89%, SD = 7%) than in Session 1 (M = 87%, SD = 8%). The main effect of ET was significant, *F*(3, 87) = 57.90, *p* < 0.001. Pair-wise comparisons revealed that accuracy was significantly different between all ETs (all *p*s < 0.05), and accuracy increased as ET increased. The main effect of Entropy was significant, *F*(5, 145) = 300.27, *p* < 0.001. Pair-wise comparisons revealed that response accuracy was significantly different between all of the Entropy conditions (all *p*s < 0.05), and decreased as the entropy increased, except between the three congruent conditions (all *p*s > 0.99). The ET × Entropy interaction was significant, *F*(15, 435) = 11.76, *p* < 0.001. Simple effects analyses showed that the effect of ET was significant under the three incongruent conditions (2:1, *F*(3, 27) = 27.14, *p* < 0.001; 4:1, *F*(3, 27) = 34.08, *p* < 0.001; 3:2, *F*(3, 27) = 19.39, *p* < 0.001) and under the 1:0 condition (*F*(3, 27) = 2.99, *p* = 0.05), but not significant under the 3:0 (*F* < 1) or 5:0 conditions (*F*(3, 27) = 2.34, *p* = 0.10). The Session × Entropy interaction was significant, *F*(5, 145) = 2.43, *p* = 0.04. Simple effects analyses showed that the effect of Entropy was significant for Session 1 (*F*(5, 25) = 199.26, *p* < 0.001) and for Session 2 (*F*(5, 25) = 113.68, *p* < 0.001). The Session × ET interaction was not significant, *F*(3, 87) = 2.37, *p* = 0.08. The three-way Session × ET × Entropy interaction was also not significant, *F* < 1.

RT increased as a function of entropy and ET (Fig. 4b). The ANOVA revealed that the main effect of Session was significant (*F*(1, 29) = 5.73, *p* = 0.023), with a shorter RT in Session 2 (M = 808 ms, SD = 34 ms) than in Session 1 (M = 857 ms, SD = 41 ms). The main effect of ET was significant (*F*(3, 87) = 25.07, *p* < 0.001). Pair-wise comparisons revealed that mean RTs were significantly different between all ETs (all *p*s < 0.05), and increased as ET increased, except between the 0.25 and 0.5 s conditions (*p* = 0.06). The main effect of Entropy was significant (*F*(5, 145) = 156.65, *p* < 0.001). Pair-wise comparisons revealed that the mean RTs were significantly different between all of the Entropy conditions (all *p*s < 0.001), and increased as a function of entropy, except between the 3:0 and 5:0 conditions (*p* > 0.99). The ET × Entropy interaction was significant (*F*(15, 435) = 16.62, *p* < 0.001). Simple effects analyses showed the effect of ET was significant for all level of Entropy (3:0, *F*(3, 27) = 3.24, *p* = 0.04; 5:0, *F*(3, 27) = 4.13, *p* = 0.02; 2:1, *F*(3, 27) = 9.89, *p* < 0.001; 4:1, *F*(3, 27) = 17.14, *p* < 0.001; and 3:2, *F*(3, 27) = 33.58, *p* < 0.001), except for the 1:0 condition (*F*(3, 27) = 2.01, *p* = 0.14). The other two-way interactions and the three-way interaction were not significant (Session × ET: *F* < 1; Session × Entropy: *F* < 1; Session × ET × Entropy: *F* < 1).

### Estimation of the capacity of cognitive control based on response accuracy

The mean *E*[*CCC*] based on each participant’s response accuracy was 3.45 bps (95% CI: 3.12 to 3.70 bps, range: 1.00 to 4.55 bps, see Fig. 4c for the histogram of the distribution of *E*[*CCC*]). Note that a large sample is needed to test whether CCC is normally distributed. The *E*[*accuracy*] in each condition based on this *E*[*CCC*] is presented in Fig. 4d, and is remarkably similar to the empirical data displayed in Fig. 4a. The empirical results revealed that the accuracy in the 4:1 conditions were higher than in the 2:1 conditions, while the grouping search predicted the reverse pattern, suggesting that there might be an alternative mental algorithm beyond the time-constrained grouping search algorithm. See further discussion about the alternative explanations in the Discussion section.

### Reliability of the estimation of capacity of cognitive control

The *E*[*CCC*] was 3.23 bps (95% CI: 2.86~3.48 bps) in Session 1 and 3.68 bps (95% CI: 3.33~4.00 bps) in Session 2. The *E*[*CCC*] values from Sessions 1 and 2 were significantly correlated across all participants (*r* = 0.75, *p* < 0.001), with a split-half reliability of 0.86, demonstrating that the estimated CCC value using the MFT-M was highly reliable.

### Model comparison

Based on the group-average response accuracy in each condition, the grouping search model with a constant chance level of 50% (*BIC* mean ± SD = 17.6 ± 3.6) fitted the empirical data better than the alternative model with a variable chance level (*BIC* = 19.0 ± 1.5, *ΔBIC = *1.33 ± 2.2). Although the *ΔBIC* was relatively small between two models, for 23 out of 30 participants, the *BIC* for the model with a constant chance level was lower than the model with a variable chance level. These results indicate that a variable chance level could not significantly improve the model fitting for the grouping search model.

The alternative model with variable processing time showed similar results as the model with the ETs as the processing time (null model): *E*[*CCC*] = 3.64 bps (95% CI: 3.38 to 3.89 bps, range: 2.01 to 4.57 bps), with a very small improvement in model fitting (*BIC* = 17.68, *ΔBIC* = −0.05 ± 0.12). However, the reliability of the estimation by this model (*r* = 0.74, *p* < 0.001, reliability = 0.85) was slightly lower than the null model. These results indicate that this complex model was not able to significantly improve the estimation. In addition, when the 2 s ET conditions was excluded from the estimation, the *E*[*CCC*] was 3.62 bps (95% CI: 3.38 to 3.88 bps, range: 1.74 to 4.68 bps), and this estimation by the model with three levels of ET was highly reliable (*r* = 0.87, *p* < 0.001, reliability = 0.93). Compared with the model with four levels of ET, this estimation was not significantly different (*t*(58) = 0.92, *p* = 0.36), but revealed better model fitting (*BIC* = 14.84 ± 2.47, *ΔBIC* = −2.90 ± 1.47), suggesting that in the future studies, the 2s ET condition can be omitted.

In addition, the grouping search model with a constant chance level (*BIC* = 17.6 ± 3.6) fitted the empirical data better than the exhaustive search model (*BIC* = 25.0 ± 3.3, Δ*BIC* = 7.4 ± 2.1) and the self-terminating model (*BIC* = 22.1 ± 3.7, *ΔBIC* = 4.5 ± 1.7). This effect was consistent across all participants. Together, these results indicate that the grouping search model was the best-fitting model.

## Discussion

### Cognitive control as a high-level process with low capacity

Compared to other sensory/motor and language domains, the CCC (estimated as 3 to 4 bps) is remarkably low. For example, capacity has been reported as ~4.3 × 10^6 ^bps for the visual system^7^, 8000 to 10000 bps for the auditory system^6^, ~40 bps for reading^4^, and ~10 bps for motor control^2^. These high-capacity domains can be considered as the transmitters of a communication system, which encode domain-specific inputs into abstract information. It is reasonable for a transmitter (e.g., a specific sensory domain) to have a high capacity because it allows us to monitor the external and internal environment automatically to prevent missing any critical signals and to make automatic responses. After encoding, a limited amount of domain-general information is passed to the conscious mind, which can be conceptualized as the receiver in the communication system. It is necessary to reduce the redundancy among inputs from lower-level domains to avoid overloading the conscious mind.

For lower-level processing (e.g., sensory encoding), the mechanism of redundancy processing (consisting of both redundancy increasing and decreasing at different neuronal levels) can be hardware-implemented without substantial involvement of cognitive control^22^,^23^,^24^ such as the unlimited-capacity preattentive processing^25^ and high-capacity iconic memory^26^. However, redundancy reduction at higher-level cognitive domains should be under the guidance of cognitive control, which optimizes the mental algorithm to improve the efficiency of information selection from vast lower-level inputs that can then be passed to the conscious mind based on goals and homeostatic demands^8^,^27^,^28^. That is, cognitive control serves as a “controller” of the encoding system as well as a “router” of the network devices to direct traffic (i.e., transmit encoded information). Thus, higher-level abstract information, such as the information in working memory, has already been encoded with an optimal code length and has substantially low redundancy. Cognitive control will further coordinate related processes and act on this higher-level information. This may explain why the capacity of sensory and motor modalities appears much greater than the capacity of cognitive control.

The low CCC may be restricted by its underlying neural mechanisms to support the conscious mind. The involvement of the cognitive control network (CCN) in uncertainty processing has been demonstrated^8^,^19^. Brain activation in regions of this network increases when the amount of information under cognitive control increases^19^,^29^,^30^,^31^,^32^ and reaches a plateau when the cognitive control system is overloaded^21^,^33^,^34^. Similar to the capacity-limited working memory system, which has been demonstrated as 7 ± 2 chunks^1^, four colors or orientations^35^, or four chunks^36^, the relatively low capacity of these higher level psychological constructs may be limited by the expensive biological cost for keeping more CCN neurons firing for processing more information^37^, or to avoid overloading the conscious mind which is capacity limited.

### Alternative models to explain the empirical performance

Our estimation was made based on a single strategy: the time-constrained grouping search. This model revealed good global fitting to the empirical data compared to two alternative strategies (exhaustive search and self-terminating search). However, other strategies are worth consideration as participants may have adopted other top-down strategies or been influenced by bottom-up effects in some specific conditions, which would lead to a poor local fitting.

Texture perception may influence the search strategy. For example, arrows in the congruent conditions can compose a special texture that can be extracted pre-attentively with extremely high capacity. However, the estimation would not be impacted because the predicted response accuracy in the congruent conditions shows ceiling effect by this texture perception model as well as by the grouping search model. In addition, some participants showed much lower accuracy in congruent conditions under 0.25 s ET, compared with other ETs, which cannot be explained by this texture perception account.

A pop-out effect could also occur and influence search strategy, especially when just a single arrow points to the minority direction (the 4:1 and 2:1 conditions). This effect might be stronger when there are more arrows pointing to the same direction that is opposite to the pop-out item, and then participants could simply make a decision by excluding the pop-out item and scanning less samples compared with the grouping search. This effect may lead to higher response accuracy in the 4:1 conditions than in the 2:1 conditions, as shown by the empirical results. However, the saliency of pop-out effect is hard to quantify and implementing it made the modeling more complicated.

Moreover, in the current grouping search model we assumed that an item-based process (from Equation 3: *n*_*a*_* = 2*^*C *×* ET*), which indicates that scanning each arrow takes a fixed amount of time. However, there is an alternative model that assumed an information-based processing, corresponding to a formula: *n*_*a*_ = *2*^(*C *×* ET*), which indicates that processing each bit of information requires a fixed amount of time. We have attempted to use this information-based model to estimate the CCC and found that the estimated CCC was similar as the item-based model, but the model fitting was poorer and the reliability was lower. Our results support the item-based model. For the information-based model, the *n*_*a*_ varies as an exponential function of ET. Therefore, a small change in ET will lead to a dramatic change in *n*_*a*_ after the inflection point. This case also seems impossible in real life performance. This may be the reason why the estimation by the information-based model was less reliable than the item-based model. Therefore, our current item-based model is more suitable for representing the underlying strategy during the task performance.

### Limitations and further questions

There are several questions still unanswered by the current study. If cognitive control serves as the central bottleneck for information processing across different sensory modalities and cognitive domains, its capacity should be modality- and domain-general^8^. Only visuo-spatial processing in the perceptual decision-making domain is involved in the MFT-M. Therefore, our conclusion cannot be directly generalized to the cognitive control of information processing in other modalities or domains. However, the cognitive load is easier to be quantified in the perceptual decision-making domain, compared with higher-level processes, such as language, emotion, and social cognition, because the information in these higher-level domains is usually more subjective and more abstract. Therefore, it is a good starting point to assess the capacity of cognitive control from the perceptual decision-making domain, and studies in this domain could offer us a preliminary theoretical understanding of the nature and magnitude of this capacity limit, and an inspiration in methods to study the capacity limit in other modality and domains. Given that cognitive control coordinates thoughts and actions under uncertainty and that the underlying mechanism of cognitive control should not be domain-dependent, we predict that for other higher-level cognitive domains, the CCC would be in the same range as what we found from this perceptual decision making task.

In addition, we did not make any prediction for RT in this study. Based on Hick’s law^38^,^39^, RT could be predicted to show a linear increase as a function of the transmitted entropy. However this transmitted information in entropy is difficult to estimate in the MFT-M. In most of the 2 s ET conditions, the mean RTs were shorter than the ET but the mean accuracy in these conditions did not reach perfect level. It may be that in some trials a VT occurred before the disappearance of the stimuli or because of speed-accuracy trade-off. The effects caused by these two possibilities are difficult to disassociate. In order to improve the efficiency of this task, future studies should fine-tune the setting of the ETs. For healthy adults, trials with long ETs can be cut without impacting the estimation of CCC. However, for patients with neuropsychiatric disorders and for children, long ET conditions may be necessary because their cognitive processing efficiency is usually lower than healthy adults. In addition, if a congruent sample is obtained before stimuli disappear, participants may keep scanning that congruent sample to reconfirm their choice of response the responses to make. The frequency of this process may be different in different conditions, and is also difficult to assess. We have attempted to address these issues by incorporating the RT and different mental algorithms in the model fitting. However, these more sophistical models did not significantly improve the estimation of CCC.

## Conclusion

In summary, we have quantified the CCC as 3 to 4 bps. Future studies are warranted to examine whether the CCC is domain- and modality-general and the relationship between cognitive control capacity and other assessments of cognitive capability such as working memory and intellectual ability. Our study may provide a foundation for the understanding of the underlying mechanisms of cognitive control and for the development of systematic and standardized measurements of individual differences in cognitive control ability, and to assess the cognitive control deficits in patients with neuropsychiatric disorders.

## Additional Information

**How to cite this article**: Wu, T. *et al*. The Capacity of Cognitive Control Estimated from a Perceptual Decision Making Task. *Sci. Rep.* **6**, 34025; doi: 10.1038/srep34025 (2016).

## Figures and Tables

**Table 1:** Means (and SE) for response accuracy (in %) and RT (in ms) for all conditions (averaged across two sessions, n = 30).

	Ratio					
	1:0	3:0	5:0	2:1	4:1	3:2
ET (s)						
	Accuracy (%)					
0.25	96.1 (0.6)	96.1 (1.3)	95.8 (1.2)	73.0 (2.0)	75.7 (2.0)	56.3 (1.7)
0.5	97.6 (0.4)	98.1 (0.6)	96.5(1.3)	79.6 (2.0)	85.8 (2.0)	63.5 (1.9)
1	97.5 (0.6)	98.0 (0.7)	98.2 (0.7)	87.5 (1.9)	91.8 (1.4)	67.1 (2.0)
2	96.7 (0.7)	98.1 (0.9)	98.2 (0.7)	89.2 (2.1)	94.0 (1.2)	75.4 (2.0)
	RT (ms)					
0.25	526 (17)	569 (21)	566 (18)	876 (39)	762 (33)	850 (40)
0.5	536 (21)	598 (26)	610 (32)	952 (35)	835 (36)	965 (43)
1	598 (48)	657 (40)	685 (46)	1041 (37)	981 (43)	1179 (46)
2	699 (90)	779 (80)	823 (89)	1217 (62)	1193 (70)	1488 (68)
